# Efficacy of concurrent treatments in idiopathic pulmonary fibrosis patients with a rapid progression of respiratory failure: an analysis of a national administrative database in Japan

**DOI:** 10.1186/s12890-016-0253-x

**Published:** 2016-06-08

**Authors:** Keishi Oda, Kazuhiro Yatera, Yoshihisa Fujino, Hiroshi Ishimoto, Hiroyuki Nakao, Tetsuya Hanaka, Takaaki Ogoshi, Takashi Kido, Kiyohide Fushimi, Shinya Matsuda, Hiroshi Mukae

**Affiliations:** Department of Respiratory Medicine, University of Occupational and Environmental Health, Japan, 1-1, Iseigaoka, Yahatanishiku, Kitakyushu City, Fukuoka 807-8555 Japan; Department of Preventive Medicine and Community Health, University of Occupational and Environmental Health, Japan, 1-1, Iseigaoka, Yahatanishiku, Kitakyushu City, Fukuoka 807-8555 Japan; Miyazaki Prefectural Nursing University, 3-5-1 Manabino, Miyazaki city, Miyazaki 880-0929 Japan; Department of Health Care Informatics, Tokyo Medical and Dental University Graduate School, 1-5-45 Yushima, Bunkyoku, Tokyo, 113-8510 Japan; Second Department of Internal Medicine, Nagasaki University School of Medicine, 1-7-1 Sakamoto, Nagasaki, 852-8501 Japan

**Keywords:** Acute exacerbation of idiopathic pulmonary fibrosis, Mechanical ventilation, Corticosteroid, Co-trimoxazole, Macrolide, Nationwide database, Acute respiratory failure

## Abstract

**Background:**

Some IPF patients show a rapid progression of respiratory failure. Most patients are treated with high-dose corticosteroids. However, no large clinical studies have investigated the prognosis or efficacy of combined treatments including high-dose corticosteroids in IPF patients with a rapid progression of respiratory failure.

**Methods:**

We enrolled IPF patients who received mechanical ventilation and high-dose corticosteroids between April 2010 and March 2013. Records were extracted from a Japanese nationwide inpatient database. We conducted a retrospective epidemiologic and prognostic analysis.

**Results:**

Two hundred nine patients receiving an average of 12.8 days of ventilatory support were enrolled. There were 138 (66 %) fatal cases; the median survival was 21 days. The short-term (within 30 days) and long-term (within 90 days) survival rates were 44.6 and 24.6 %, respectively. The average monthly admission rate among the IPF patients with the rapid progression of respiratory failure in the winter was significantly higher than that in spring (*p* = 0.018). Survival did not differ to a statistically significant extent in the different geographic areas of Japan. Survivors were significantly younger (*p* = 0.002) with higher rates of mild dyspnea on admission (*p* = 0.012), they more frequently underwent bronchoscopy (*p* < 0.001), and received anticoagulants (*p* = 0.027), co-trimoxazole (*p* < 0.001) and macrolide (*p* = 0.02) more frequently than non-survivors. A multivariate logistic analysis demonstrated that two factors were significantly associated with a poor prognosis: >80 years of age (OR = 2.94, 95 % Cl 1.044–8.303; *p* = 0.041) and the intravenous administration of high-dose cyclophosphamide (OR = 3.17, 95 % Cl 1.101–9.148; *p* = 0.033). Undergoing bronchoscopy during intubation (OR = 0.25, 95 % Cl 0.079–0.798; *p* = 0.019) and the administration of co-trimoxazole (OR = 0.28, 95 % Cl 0.132–0.607; *p* = 0.001) and macrolides (OR = 0.37, 95 % Cl 0.155–0.867; *p* = 0.033) were significantly associated with a good prognosis. The dosage of co-trimoxazole significantly correlated with survival.

**Conclusions:**

Co-trimoxazole and macrolides may be a good addition to high-dose corticosteroids in the treatment of IPF patients with a rapid progression of respiratory failure.

## Background

Idiopathic pulmonary fibrosis (IPF) is a progressive parenchymal lung disease with an estimated median survival of 3–5 years from the time of diagnosis [1, 2]. The disease behavior in patients with IPF is usually diverse, with some IPF patients showing the rapid progression of respiratory failure [3, 4]. The mortality rate in IPF patients with severe respiratory failure who require a ventilator is around 90 % [5].

Most of the severe IPF patients who show rapid progression of respiratory failure receive high-dose corticosteroids [3, 6]. The 2011 international evidence-based guideline indicates that it as weak positive recommendation [2] in patients with definite or suspected [7] acute exacerbation of IPF (AE-IPF). Thus far, however, there have been no large clinical data sets to investigate the prognosis of patients with AE-IPF who receive ventilator treatment and high-dose corticosteroids. In addition, patients with AE-IPF are pathologically heterogeneous [8], and the appropriate treatment strategy for AE-IPF patients is not fully understood. Recent treatments for patients with AE-IPF include new agents, such as thrombomodulin [9, 10] and new ventilator setting strategies that aim to avoid valotrauma [11, 12]. Such treatments show some promise in their potential to improve the survival rate.

The aim of the present study was to evaluate the epidemiology and prognosis of IPF patients with severe rapid progression of respiratory failure who required ventilator support in Japan, using a large, contemporary, and comprehensive Japanese clinical database, and to explore effective combined treatment options that include the administration of high-dose corticosteroids.

## Methods

### Data source

We used the Japanese Diagnosis Procedure Combination (DPC) database, a nationwide inpatient database, to collect patient data. The details of the DPC inpatient database have been described previously [13]. Briefly, the DPC is a case-mix patient classification system which includes the clinical data and information on the date of admission, the charges, and the quantity of medical care items. The database is linked with a lump-sum per-diem payment system. Data from hospitals including all 82 university hospitals in Japan are gathered and merged into a standardized electronic format by the Japanese Ministry of Health, Labour, and Welfare. The database covers more than 1,500 acute care hospitals located throughout Japan and about 50,000 hospital beds. It represents approximately 50 % of all of the acute care hospitalizations during the same period in Japan. The database includes the main diagnoses, comorbidities present at admission and in-hospital complications as defined in the International Classification of Diseases and Related Health Problems, 10th Revision (ICD-10) codes and text data (in Japanese). The database also includes the following data: patient age and sex; height; body weight; Fletcher, Hugh-Jones (F, H-J) classification; Brinkman Index; drug use; diagnostic and therapeutic procedures; date of admission; length of stay; status at discharge; and the unique identifiers of the hospitals. Attending physicians are obliged to record the diagnoses for each patient at discharge with reference to medical charts to optimize the accuracy of the recorded diagnoses. All of the data were anonymously collected in the database, thus the requirements for informed consent were waived. This study was approved by the Ethics Committee of Tokyo Medical and Dental University, Tokyo, Japan (approval number 788).

### Patient selection and data retrieval

**Fig. 1:**
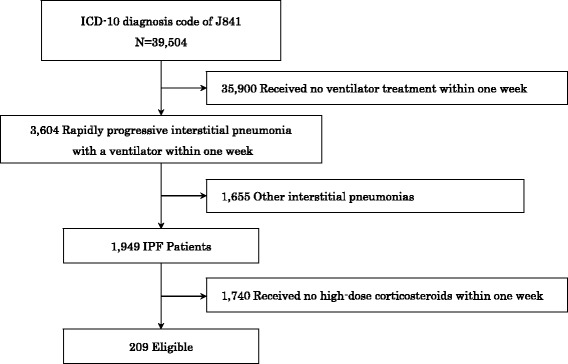
Sample Selection

From the total of 39,504 patients who were admitted to the hospitals with a principal diagnosis of other interstitial pulmonary diseases with fibrosis (ICD-10 code J841) and who were discharged between April 2010 and March 2013 patients, we excluded 35,900 patients who did not receive invasive mechanical ventilation within one week after admission because we intended to only evaluate IPF patients with the rapid progression of respiratory failure. Next, non-IPF patients (*n* = 1,655) were excluded based on the text data, followed by patients who were not treated with high-dose corticosteroids (methylprednisolone: ≥500 mg, daily) within one week after admission (*n* = 1,740). The final study population included 209 patients (Fig. 1). These patients did not include any patients with viral, fungal or infectious bacterial pulmonary diseases as the main diagnosis or as comorbidities at the time of admission.

### Definition of variables

Subjective respiratory symptoms, such as cough and dyspnea, were measured using the F, H-J classification [14]. The institution criteria authorized by The Japanese Respiratory Society were used for defining a respiratory specialized hospital. The seasons on admission were defined as follows: “spring,” from March to May; “summer,” from June to August; “fall,” from September to November; and “winter” from December to February.

### Statistical analyses

A poisson multivariable regression analysis was used to evaluate the differences in the admission rates between the seasons to adjust for the effect of the fiscal year. The chi-squared test or Fisher's exact test were used as appropriate to analyze the differences in the clinical features between survivors and non-survivors. To analyze the prognostic factors for overall survival, a univariate logistic regression were initially used to select statistically significant clinical characteristics (sex, age, performing bronchoscopy and an F, H-J classification) and to evaluate each treatment effect (the variables included treatments were applicable at least 10 % of all patients). The final multivariate logistic regression models with backward elimination were also used including the predictors (sex, age, significant clinical characteristics and the treatment). Odds ratios (OR) and 95 % confidence intervals (CI) were calculated. *P* values of <0.05 were considered to be statistically significant. All calculations were performed using the STATA 13 software program (Stata, College Station, TX).

## Results

### Patient characteristics

The clinical characteristics of the patients are shown in Table 1. The mean (standard deviation; SD) age was 72.3 (9.6) years and 82.3 % of the patients were men. The average monthly admission rate among the IPF patients with the rapid progression of respiratory failure in the winter was significantly higher than that in spring (*p* = 0.018), but was not significantly different to the rates in summer (*p* = 0.065) and fall (*p* = 0.379). The rate of emergent transfer was 48.3, and 82.8 % of the patients were admitted to specialty hospitals. Ventilatory support was provided for an average of 12.8 days.

**Table 1:** The clinical characteristics of the participants

	*N* (%)	Mean ± SD
Patients	209	
Age, years	209	72.3 ± 9.6
<60	17 (8.1)	
60–69	52 (24.9)	
70–79	98 (46.9)	
≥80	42 (20.1)	
Male	172 (82.3)	
BMI, kg/m^2^	178	22.3 ± 3.8
<18.5	27 (15.2)	
18.5–25	114 (64.0)	
>25	37 (20.8)	
Brinkman Index	184	599.5 ± 675.7
0	68 (37.0)	
1–800	45 (24.5)	
>800	71 (33.6)	
F, H-J Classification scale	166	
1	2 (1.2)	
2	4 (2.4)	
3	7 (4.2)	
4	28 (16.9)	
5	125 (75.3)	
Season		
Spring	48 (23.0)	
Summer	34 (16.2)	
Fall	56 (26.8)	
Winter	71 (34.0)*	
Specialty hospital		
Yes/No	173 (82.8)/36 (17.2)	
Arrival by ambulance		
Yes/No	101 (48.3)/108 (51.7)	

*Abbreviations*: *BMI* body mass index, *F*, *H-J* Fletcher, Hugh-Jones. Values are given as mean ± SD or n (%). The total dose was not equal to 209 because there were missing values in the data file. *Significantly different in comparison to spring (*p* = 0.018, poisson regression)

### Outcome

**Fig. 2:**
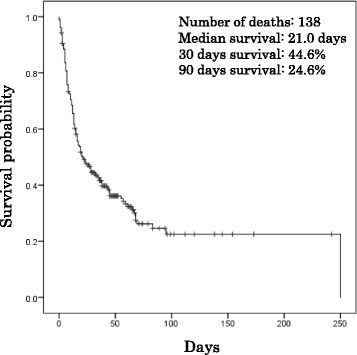
Kaplan-Meier estimates of survival from the time of admission for all of the IPF patients

**Fig. 3:**
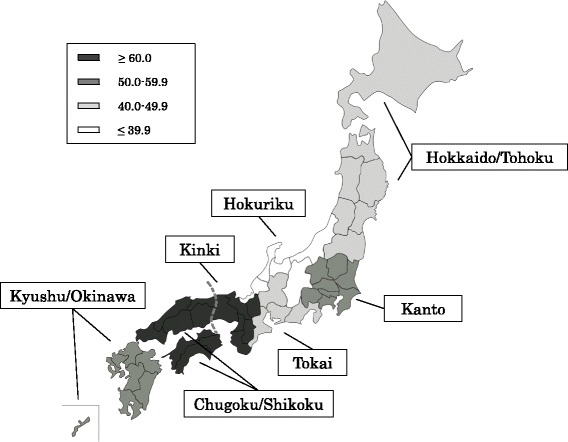
The definition of the regions in Japan and short-term mortality

The number of patients with fatal outcomes was 138 of 209 (66 %) during the observation period. The median survival period was approximately 21 days after admission. The short-term (within 30 days) and long-term (within 90 days) survival rates were 44.6 and 24.6 %, respectively (Fig. 2). The Hokuriku area had the highest rate of short-term survival (60 %), however, this rate was not significantly different from other areas (Fig. 3). Bronchoscopy and tracheostomy was performed in 20 (9.6 %) and 16 (7.7 %) patients during hospitalization, respectively. The patient characteristics of the survivors (patients who discharged alive) and the non-survivor (patients who died in the hospital) are summarized in Table 2. The survivors were younger (*p* = 0.002), with mild symptoms of dyspnea on admission (*p* = 0.012), higher rates of bronchoscopy during intubation (*p* < 0.001), anticoagulant (unfractionated and low-molecular-weight heparin) use (*p* = 0.027), co-trimoxazole use (*p* < 0.001) and macrolide use (*p* = 0.020) in comparison to non-survivors. Twenty-seven of 71 (38 %) survivors who were discharged from the hospitals received home oxygen therapy.

**Table 2:** The comparison of the clinical features of survivors and the non-survivors

	Survivors	Non-survivors	*p*-value
Patients	71	138	
Age, years	69.3 ± 10.2	73.8 ± 8.8	0.002
Male	56 (78.9)	116 (84.1)	0.352
BMI, kg/m^2^	22.2 ± 3.9	22.3 ± 3.8	0.795
Brinkman Index			
0/1–800/>800	26/14/25	42/31/46	0.719
F, H-J Classification scale			
1/2/3/4/5	1/3/3/16/42	1/1/4/12/83	0.012
Specialty hospital care	60 (84.5)	113 (81.9)	0.634
Ambulance transfer	32 (45.1)	69 (50.0)	0.499
Performing bronchoscopy	14 (19.7)	6 (4.4)	<0.001
Treatment regimen use			
Sivelestat	37 (52.1)	67 (48.9)	0.626
Diuretic drug	37 (52.1)	65 (47.1)	0.492
Anticoagulant therapy	37 (52.1)	50 (36.2)	0.027
Immunosuppressive therapy	30 (42.3)	45 (32.6)	0.169
Intravenous high-dose cyclophosphamide	9 (12.7)	23 (16.7)	0.448
PMX	4 (5.6)	9 (6.5)	0.801
Recombinant human soluble thrombomodulin	3 (4.2)	6 (4.4)	0.967
Antibiotic therapy	71 (100)	135 (97.8)	0.211
β-Lactams	60 (84.5)	116 (84.1)	0.933
Co-trimoxazole	56 (78.9)	59 (42.8)	<0.001
Quinolones	38 (53.5)	65 (47.1)	0.379
Macrolides	23 (32.4)	25 (18.1)	0.020
Tetracycline	6 (8.5)	12 (8.7)	0.952
Anti-MRSA antibiotics	6 (8.5)	11 (7.8)	0.904
Clindamycin	3 (4.2)	2 (1.5)	0.214
Aminoglycoside	2 (2.8)	1 (0.7)	0.228
Others	5 (7.0)	7 (5.1)	0.562

Data are presented as mean ± SD or n (%), unless otherwise indicated. Definition of abbreviations: BMI = Body Mass Index, F, H-J = Fletcher, Hugh-Jones, PMX = Direct hemoperfusion with polymyxin B-immobilized fiber, MRSA = Methicillin-resistant *Staphylococcus aureus*. The total dose was not equal to 209 because there were missing values in the data file

### Prognostic factors

A univariate logistic analysis indicated two significant risk factors for in-hospital mortality: ≥80 years of age (*p* = 0.033) and an F, H-J classification scale of 5 (*p* = 0.012), whereas the performance bronchoscopy, and the use of anticoagulants, co-trimoxazole and macrolides were correlated with a good prognosis (Table 3). The multivariate logistic analysis demonstrated that two variables were significantly correlated with in-hospital mortality: ≥80 years of age (*p* = 0.041) and the intravenous administration of high-dose cyclophosphamide (≥100 mg, daily) (*p* = 0.033). In contrast, the following variables were significantly correlated with a good prognosis: the performance of bronchoscopy during intubation (OR = 0.25, 95 % Cl 0.079–0.798; *p* = 0.019), and the administration of co-trimoxazole (OR = 0.28, 95 % Cl 0.132–0.607; *p* = 0.001) and macrolides (OR = 0.37, 95 % Cl 0.155–0.867; *p* = 0.022). The mortality rates of the patients who were treated in specialty and non-specialty hospitals did not differ to a statistically significant extent.

**Table 3:** Prognostic factors for survival

Variable		Univariate logistic analysis			Multivariate logistic analysis		
	*N* (%)	OR	95 % Cl	*p*-value	OR	95 % Cl	*p*-value
Age, years	209						
<60	17 (8.1)	ref			ref		
60–69	52 (24.9)	1.12	0.374–3.362	0.839			
70–79	98 (46.9)	1.83	0.647–5.196	0.254			
≥80	42 (20.1)	3.78	1.110–12.858	0.033	2.94	1.044–8.303	0.041
Male	172 (82.3)	1.41	0.681–2.930	0.354			
BMI, kg/m^2^	178						
<18.5	27 (15.2)	ref					
18.5–25	114 (64.0)	1.43	0.604–3.388	0.415			
>25	37 (20.8)	1.13	0.409–3.117	0.814			
Brinkman Index	184						
0	68 (37.0)	ref					
1–800	45 (24.5)	1.37	0.617–3.046	0.439			
>800	71 (33.6)	1.14	0.571–2.271	0.712			
F, H-J Classification scale	166						
1–4	41 (24.7)	ref			ref		
5	125 (75.3)	2.53	1.229–5.187	0.012	2.13	0.931–4.856	0.073
Specialty hospital care	173 (82.8)	0.83	0.382–1.799	0.635			
Ambulance transfer	101 (48.3)	1.22	0.686–2.164				
Performing bronchoscopy	20 (9.6)	0.19	0.068–0.506	0.001	0.25	0.079–0.798	0.019
Sivelestat	104 (49.8)	0.87	0.489–1.538	0.626			
Diuretic drug	102 (48.8)	0.82	0.461–1.451	0.493			
Anticoagulant therapy	87 (41.6)	0.52	0.292–0.933	0.028			
Immunosuppressive therapy	75 (35.9)	0.66	0.366–1.193	0.17			
Intravenous high-dose cyclophosphamide	32 (15.3)	1.38	0.601–3.160	0.449	3.17	1.101–9.148	0.033
PMX	13 (6.2)	1.17	0.347–3.935	0.801			
Recombinant human soluble thrombomodulin	9 (4.3)	1.03	0.250–4.247	0.967			
Antibiotic therapy	206 (98.6)	–	–				
β-Lactams	176 (84.2)	0.97	0.440–2.126	0.933			
Co-trimoxazole	115 (55.0)	0.20	0.103–0.388	<0.001		0.28	0.132–0.607
Quinolones	103 (49.3)	0.77	0.436–1.372	0.38			
Macrolides	48 (23.0)	0.46	0.239–0.893	0.022		0.37	0.155–0.867
Tetracycline	18 (8.6)	1.03	0.370–2.875	0.952			
Anti-MRSA antibiotics	17 (8.1)	0.94	0.332–2.651	0.904			
Clindamycin	5 (2.4)	0.33	0.054–2.042	0.235			
Aminoglycoside	3 (1.4)	0.25	0.022–2.826	0.264			
Others	12 (5.7)	0.71	0.216–2.307	0.564			

Data are presented as n (%), unless otherwise indicated. Definition of abbreviations: *BMI* body mass index, *F, H-J* Fletcher, Hugh-Jones, *PMX* Direct hemoperfusion with polymyxin B-immobilized fiber, *MRSA* Methicillin-resistant *Staphylococcus aureus*

### The relationship between survival and co-trimoxazole dosage

**Fig. 4:**
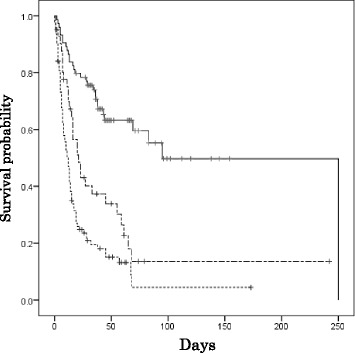
Kaplan-Meier survival estimates according to the different doses of co-trimoxazole. A log-rank test revealed significant differences between all of the groups. High-dose (solid line, 74 patients), low-dose (broken line, 41 patients), none (dotted line, 94 patients)

We examined the difference in the survival rates of patients who were treated with by co-trimoxazole tablets (sulfamethoxazole [400 mg] and trimethoprim [80 mg]) or the equivalent dose of co-trimoxazole granules and injections. Figure 4 represents the relationship between the survival rates of three groups of patients who were treated with different doses of co-trimoxazole. The doses were defined as follows: high dose (solid line; ≥6 tablets daily, *n* = 74), low dose (broken line; 1–5 tablets daily, *n* = 41) and no co-trimoxazole (dotted line; *n* = 94). Co-trimoxazole treatment in the low-dose and high-dose groups was initiated an average of 7.9 and 8.3 days after admission, respectively. The survival of the patients in the high-dose group was significantly longer than that in the low-dose and no co-trimoxazole groups (log rank *p* < 0.001). The survival of the patients in the low-dose group was also significantly longer than that in the no co-trimoxazole group (log rank *p* = 0.009) (Fig. 4).

## Discussion

Thus far, there have been no large data sets on the prognosis and prognostic factors in IPF patients with rapid progression of respiratory failure. The data of the present study, which were extracted from a nation-wide Japanese epidemiological database show, for the first time, the current prognosis of IPF patients with rapid progression of respiratory failure who receive treatment with high-dose corticosteroids. Treatments with co-trimoxazole and macrolides were significantly associated with a good prognosis and are considered to be effective when administered in combination with high-dose corticosteroids. The performance of bronchoscopy during ventilatory support was also correlated with a good prognosis. Conversely, the intravenous administration of high-dose cyclophosphamide was significantly associated with a poor prognosis. Our findings suggest that these managements may improve the morbidities associated with severe rapidly progressive of IPF.

Using a large, nationally representative Japanese database allowed us to investigate the prognostic factors for in-hospital mortality in IPF patients with rapid progression of respiratory failure. The application of mechanical ventilation in IPF patients with respiratory failure is considered to be a “weak” recommendation [2]. The 90-day mortality rate of patients with severe rapidly progressive IPF who received mechanical ventilation (75.4 %) was lower than the previously reported rate (approximately 90 %) [5, 15]. Our results indicate that in such patients, the provision of mechanical ventilation may still be controversial. Similarly to stable patients with IPF [16], IPF patients who were older than 80 years of age showed a very poor prognosis in the present study. According to our data, ventilatory support may not be recommended in these patients. Similar to a previous report [17], a significantly higher number of patients were admitted due to IPF with rapid progression of respiratory failure in winter than in spring; however, there were no location-based differences in Japan. In contrast with stable patients with IPF [18], no differences of mortality were observed according to whether or patients were admitted to specialty or non-specialty hospitals.

Although there have only been limited data on the role of bacterial infection in IPF patients, recent reports suggest the high importance of infectious causes and the importance of the progression of IPF [19, 20] in the development of AE-IPF [21]. In cases where the exclusion of infectious causes was insufficient (e.g. when they were diagnosed by the analysis of endotracheal aspirate or bronchoalveolar lavage fluid), they were considered as cases of suspected AE-IPF. It has been reported that the prognosis of patients with suspected AE-IPF is not significantly different to that of patients with definite AE-IPF [7]. The data in the present study showed that the performance of bronchoscopy might be related to a better prognosis (Table 3). The majority of AE-IPF patients received empiric antimicrobial treatments, which targeted common respiratory pathogens (although there has been no data to support their use in AE-IPF patients) [22]. The infectious causes of respiratory failure can easily missed, even when several microbiological tests are performed for clinical reasons: in the treatment of such patients it is not usually possible to wait for microbiological results, and antimicrobial treatments are considered to be low-risk. The results of the present study suggest that antibiotic treatments (other than co-trimoxazole and macrolides) may not contribute to the survival of AE-IPF patients with respiratory failure. This study also showed a trend towards poor survival in patients who were treated with immunosuppressants, especially intravenous high-dose cyclophosphamide. Currently, there is no strong evidence to support the use of immunosuppressants in AE-IPF patients with respiratory failure, and further studies will be needed to evaluate the role of immunosuppressants when they are administered in combination with high-dose corticosteroids.

Several reports have described the relationship between the prognosis of IPF patients and the administration of co-trimoxazole or macrolides. Shimizu et al. hypothesized that the specific role of co-trimoxazole was linked to a high prevalence of *Pneumocystis jiroveci* colonization among patients with stable IPF [23]. Shulgina et al. also reported that the addition of co-trimoxazole therapy to the standard treatment for stable patients with fibrotic idiopathic interstitial pneumonia resulted in improved quality of life and a reduction in mortality [24]. Huie et al. investigated the potential role of infection in the exacerbation of acute respiratory symptoms in patients with IPF [25] and showed *P. jiroveci* may be associated with the onset of AE-IPF. Our data indicated that the administration of co-trimoxazole mortality in IPF patients with rapid progression of respiratory failure, not only at the treatment dose for *P. jiroveci* but also at lower doses. This favorable effect of co-trimoxazole might, in addition to its antimicrobial activity against *P. jiroveci*, may be due to its anti-inflammatory effect: co-trimoxazole might have reduced the neutrophil-derived oxidative stress [26]. Macrolides have also been reported to have anti-inflammatory effects [27, 28]. Kawamura et al. reported that azithromycin was associated with improved outcomes in patients with acute exacerbation of chronic fibrosing interstitial pneumonia [29]. The administration of macrolides and co-trimoxazol may therefore increase the survival rate in patients with AE-IPF.

The present study is associated with several limitations. First, this study was a retrospective observational study. However, the analysis of the large data set of the DPC database system allowed us to perform our study in a large patient population. Tzilas et al. reported the weakness of the ICD coding system [30]; however, in addition to the ICD data our study also used the text data that were recorded in the DPC database system to clarify the physicians’ diagnoses. Second, several clinical variables were not obtained, including the medication data and the patients’ respiratory function before admission, and the results of bacteriological tests. However, we only enrolled IPF patients with rapid progression of respiratory failure in whom a ventilator was used on admission to ensure that the severity of IPF in our study population was uniform at the start of the observation period. Third, the DPC database system can only record in-hospital data, thus the data of the patients who were discharged from the DPC hospitals was not available and the contribution of their medications to survival may be underestimated. Finally, it is uncertain whether the regional clinical pathway would work the same way in other countries where health systems and policies differ from those in Japan. The effectiveness of the clinical pathway has been shown to be inconsistent in different areas and further studies are necessary to examine the applicability of this system to other countries.

## Conclusion

We herein showed the epidemiology and prognosis of IPF patients with rapid progression of respiratory failure in recent years using a national administrative database in Japan. The performance of bronchoscopy during intubation, and the administration of co-trimoxazole and macrolides were significantly good prognostic factors. The concomitant use of co-trimoxazole in addition to high-dose corticosteroids may improve survival in IPF patients with rapid exacerbation of respiratory failure. Further clinical trials are necessary to verify the findings of the present study.

## Key messages

- Rapid progression of respiratory failure in IPF patients was frequently seen in the winter. Older age and a higher grade of dyspnea on admission were poor prognostic factors. In addition, the prognosis of the patients did not differ in relation to the geographic area of Japan or in patients who were treated in a specialized hospital.
- The results of this study indicate that the prognosis of patients who underwent a bronchoscopic examination was better prognosis than those who did not; however, the performance of bronchoscopy in AE-IPF patients might be controversial.
- Regarding the treatment of IPF patients with rapid progression of respiratory failure, the use of high-dose corticosteroids in combination with macrolides and co-trimoxazole may lead to a better prognosis. However, prospective randomized controlled trials are necessary to elucidate the clinical effects of these agents.

## Abbreviations

AE-IPF, Acute exacerbation of IPF; CI, Confidence Intervals; DPC, Diagnosis Procedure Combination; F, H-J, Fletcher, Hugh-Jones; ICD-10, International Classification of Diseases and Related Health Problems, 10th Revision; IPF, Idiopathic Pulmonary Fibrosis; OR, Odds ratios
